# Physiological and Biochemical Responses of Tomato Plants Grafted onto *Solanum pennellii* and *Solanum peruvianum* under Water-Deficit Conditions

**DOI:** 10.3390/plants10112236

**Published:** 2021-10-20

**Authors:** Flávia Maria Alves, Madhumita Joshi, Desire Djidonou, Vijay Joshi, Carlos Nick Gomes, Daniel Ivan Leskovar

**Affiliations:** 1 Departamento de Agronomia, Universidade Federal de Viçosa, Viçosa 36570-000, MG, Brazil; flaviamarialves@gmail.com (F.M.A.); carlos.nick@ufv.br (C.N.G.); 2 Texas A&M AgriLife Research and Extension Center, Texas A&M University, Uvalde, TX 78801, USA; Madhumita.Joshi@ag.tamu.edu (M.J.); Vijay.Joshi@ag.tamu.edu (V.J.); 3 College of Agricultural Sciences and Natural Resources, Texas A&M University, Commerce, TX 75428, USA; desire.djidonou@tamuc.edu

**Keywords:** grafting, wild relative species, gas exchange

## Abstract

Grafting using suitable rootstocks mitigates the adverse effects caused by environmental stresses such as water deficit in the tomato crop. *Solanum pennellii* and *Solanum peruvianum*, the wild relatives of tomato, are used as rootstocks due to their tolerance to water deficit and soil-borne diseases. This study focused on evaluating physiological and biochemical responses of tomato plants grafted onto *S. pennellii* and *S. peruvianum* rootstocks during water deficit. The commercial tomato cultivar ‘HM 1823’ (HM) either self-grafted (HM/HM) or grafted onto *S. pennellii* (HM/PN), *S. peruvianum* (HM/PR), and ‘Multifort’ (HM/MU) rootstocks were subjected to water-deficit stress by withholding irrigation for eight days. The performance of the grafted plants under water deficit was evaluated using physiological and biochemical parameters in vegetative tissues of the grafted plants. Plants grafted using *S. pennellii* (PN) and *S. peruvianum* (PR) rootstocks showed higher values of water potential (Ψw), relative water content (RWC), net photosynthetic rate (A), and leaf water use efficiencies (WUE) compared to HM, HM/HM, and HM/MU. Plants grafted onto tomato wild relatives showed the lowest malondialdehyde (MDA) and proline content. This study demonstrated that the rootstocks of wild tomato relatives reduced the effect of water deficit to a greater extent through better physiological, metabolic, and biochemical adjustments than self-grafting plants.

## 1. Introduction

Tomato (*Solanum lycopersicum*) is one of the most widely cultivated vegetable crops, with 5 million hectares harvested and over 180 million tons produced worldwide in 2019 [1]. However, field-grown tomato is sensitive to drought and has been reported to sustain yield losses under water-deficit conditions [2]. Water deficit caused by drought is one of the most critical abiotic stresses impacting plant growth, yield, and quality [3]. This abiotic stress occurs when water loss by the plant exceeds the capacity of water absorption by the roots for long enough to cause morphological, physiological, and biochemical changes [4].

To cope with drought stress, plants have developed several defensive mechanisms, which include changes in root architecture; more robust root systems; the production of epidermal wax; the modulation of stomatal conductance to reduce transpiration; the production of osmolytes; scavenging reactive oxygen species (ROS); and the mobilization of stress-related hormones [5,6,7]. Understanding these mechanisms of drought tolerance at the morphological, physiological, biochemical, and molecular levels is critical for crop improvement.

Yield reduction from water deficit, which can reach up to 50%, is dependent on the stress frequency, severity, and duration [8,9]. This yield reduction is observed because commercial tomato cultivars inherently lack proper physiological and biochemical traits linked with tolerance to water deficit [10]. Natural biodiversity provides an opportunity to use wild tomato species, which represents the resource for biotic and abiotic stress tolerance.

In this regard, the use of grafting in commercial tomato production to increase WUE has been widely discussed. Studies have reported the efficiency of rootstocks in reducing the effects of water deficit on the scion by altering plant water homeostasis and improving WUE in tomatoes. It has been demonstrated that the genotype of the rootstock regulates the scion transpiration rate and its acclimation to water-deficit conditions through modulation of genetic architectures [11]. Positive effects of the rootstock’s root system in maintaining the leaf relative water content (RWC) through enhancing the absorption of water and nutrients have been shown in tomatoes [12,13,14]. The influence of rootstock was also noted for the osmotic adjustment and accumulation of a range of osmolytes, such as proline, GABA, and other amino acids in pepper and tomato-grafted plants [14,15,16].

Moreover, plants subjected to water deficit tend to overproduce ROS. However, tomato-grafted plants under water deficit activate and/or modulate the antioxidant defense system, and this has played an essential role in reducing damage by lipid peroxidation, making these plants tolerant to water deficit [14]. Efficiency in water use and reducing water loss can maintain the photosynthesis rate in grafted plants under water deficit by allowing for plant growth and yield [12,16,17].

Grafting also provides opportunities to utilize the natural genetic variability present in wild-type relatives for root-specific traits to improve the performance of commercial tomato varieties. By using recombinant inbred lines (RILs) derived from wild-type tomato species *Solanum pimpinellifolium*, they have shown that tomato WUE can be improved by rootstock-derived hormonal-signaling under water stress conditions.

Sources of genetic variability for abiotic stress tolerance have been reported among and within wild tomato species, such as: *Solanum pimpinellifolium, Solanum peruvianum, Solanum cheesmaniae, Solanum habrochaites, Solanum chmielewskii*, and *Solanum pennellii* [18]. However, the introgression of genetic traits from distant wild relatives to cultivated tomatoes by conventional breeding is challenging because of cross-ability barriers and linkage drag. The grafting technique is a valuable tool and an integrative reciprocal process, where both rootstock and scion can influence the stress tolerance of grafted plants [19,20]. *S. pennellii* (Corr.) D’Arcy and *S. peruvianum* (L.) Mill. are two wild tomato relatives considered tolerant to water deficit [21] and might be helpful as rootstocks to obtain drought-tolerant grafted plants.

The wild relative *S. pennellii* is native to the Andean area of South America and is evolutionarily adapted to arid conditions [22,23]. The distribution of *S. peruvianum* is mostly in coastal central/southern Peru and northern Chile. This species is commonly found growing in arid, sandy, or rocky dry washes [24]. The wild type *S. pennellii* was widely used and studied for its drought tolerance characteristic. It has been suggested that the inherent ability of *S. pennellii* to increase water use efficiency and its morphological characteristics, such as it thick waxy leaves and acyl-sugars in the trichomes, help to minimize transpiration under drought conditions [25]. To investigate the molecular mechanism of drought tolerance, several studies exploited the RILs of *S. pennellii* and discovered both the drought-responsive genes and QTLs by transcriptional profiling and biochemical pathway analysis [26].

This study was designed to evaluate the capacity of *S. pennellii* and *S. peruvianum* rootstock to enhance drought tolerance in grafted tomato plants. We hypothesized that tomato plants grafted onto *S. pennellii* and *S. peruvianum* are more efficient in water use and have less oxidative damage than non-grafted or tomato plants grafted onto other rootstocks under water-deficit stress. To test these two hypotheses, we measured morphological, physiological, and biochemical traits associated with drought tolerance to evaluate the WUE of grafted plants.

## 2. Results and Discussion

**Figure 1:**
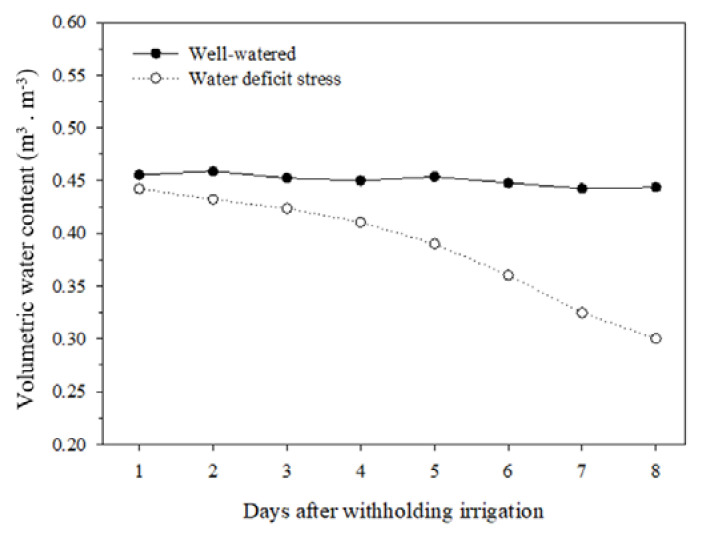
Volumetric water content in well-watered and water-deficit stress conditions for 8 days.

Volumetric water content (VWC) was held constant in both treatments by daily irrigation until 30 days after transplanting. Thereafter, water was withheld for 8 days in plants subjected to water-deficit stress conditions, while well-watered pants received daily irrigation. Figure 1 shows the downtrend in VWC for plants under deficit stress, which reached a 32.1% reduction compared to the well-watered plants.

The interaction of water regimes and grafting/non-grafted combinations was significant (*p* < 0.05) for all plant physiological and biochemical traits measured, except for leaf transpiration rate (Table 1).

### 2.1. Plant Water Status

**Figure 2:**
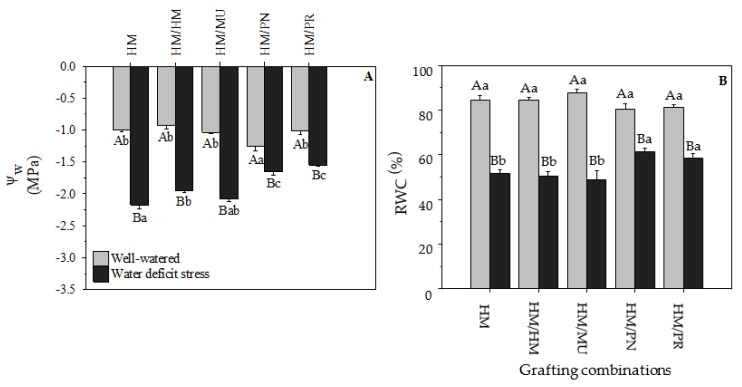
(**A**) Water potential (Ψ_w_) and (**B**) relative water content (RWC) of five grafting combinations under well-watered and water-deficit stress conditions. Capital letters denote significant differences between water regimes within each graft combination and lower-case letters denote significant differences among grafting combinations within each water regime according to the LSD test (*p* ≤ 0.05). Columns are mean ± standard error. Note: HM = non-grafted HM 1823; HM/HM = HM 1823 self-grafted; HM/MU = HM 1823 grafted onto Multifort; HM/PN = HM 1823 grafted onto *S. pennellii*; and HM/PR = HM 1823 grafted onto *S. peruvianum*.

The HM/PN combination had the lowest leaf water potential (−1.26 MPa) in the control treatment, while the other grafting and non-grafted combinations showed no significant difference. Under water stress conditions, the leaf water potential of HM/PN (−1.65 MPa) and HM/PR (−1.55 MPa) was significantly higher in comparison to the HM/MU (−2.08 MPa), HM/HM (−1.95 MPa), and HM (−2.17 MPa; Figure 2A). The non-grafted had the lowest leaf water potential under water-deficit stress conditions. The HM/PR and HM/PN grafting combinations showed the lowest reductions between the two water regimes (34.8% and 23.8%, respectively). Low water availability in the soil induces a decrease in leaf water content and leaf water potential [27,28].

In the stress condition, HM, HM/HM, and HM/MU showed the lowest leaf water potential values. This suggests that they are more sensitive to water deficit when compared to HM/PR and HM/PN. Leaf water potential was used to quantify water-deficit tolerance in several cultivated plants [29,30,31].

The RWCs were not significantly different between the grafting and non-grafted combinations under well-watered conditions, but a significant reduction in RWC was observed under stress. RWCs decreased in HM, HM/HM, HM/MU, HM/PN, and HM/PR by 39.0%, 39.9%, 44.2%, 24.9%, and 27.9% compared with their controls, respectively (Figure 2B). The grafting combinations that showed the highest RWCs under water-deficit stress were HM/PN (61.2%) and HM/PR (58.7%).

Under water deficit, the RWC is reduced, damaging cell membranes and affecting plant physiological responses [32,33]. *S. pennellii* showed higher RWC than *S. lycopersicum* under water-deficit conditions [32]. Several studies have demonstrated the high correlation between RWC and the ability of plant genotypes to tolerate water deficit [33,34]. The maintenance of RWC was considered as one of the best criteria to identify and classify tolerant and sensitive genotypes [34,35].

### 2.2. Gas Exchange Measurements

**Figure 3:**
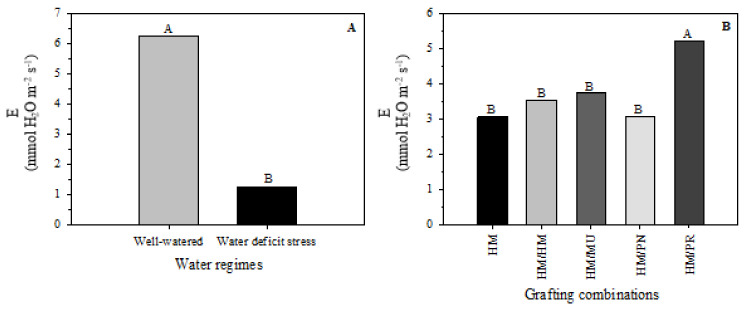
Leaf transpiration rate (E) of five grafting combinations (**B**) under well-watered and water-deficit stress conditions (**A**). Different letters denote significant differences between the water regimes and graft combinations according to the LSD test (*p* ≤ 0.05). Note: HM = non-grafted HM 1823; HM/HM = HM 1823 self-grafted; HM/MU = HM 1823 grafted onto Multifort; HM/PN= HM 1823 grafted onto *S. pennellii*; and HM/PR = HM 1823 grafted onto *S. peruvianum*.

The average leaf transpiration rate was 6.253 mmol H_2_O m^−2^ s^−1^ for well-watered plants compared to 1.252 mmol H_2_O m^−2^ s^−1^ in water-deficit stress plants, representing an average reduction of 80% (Figure 3A). HM/PR had the highest leaf transpiration rate (5.20 mmol H_2_O m^−2^ s^−1^). Non-grafted plants (3.07 mmol H_2_O m^−2^ s^−1^), HM/HM (mmol 3.54 H_2_O m^−2^ s^−1^), HM/MU (3.75 H_2_O m^−2^ s^−1^), and HM/PN (3.20 H_2_O m^−2^ s^−1^) showed no statistical difference in this variable (Figure 3B).

**Figure 4:**
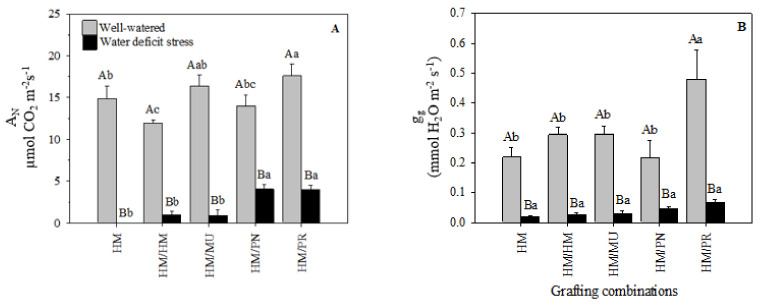
(**A**) Net photosynthetic rate (A_N_) and (**B**) stomatal conductance (g_s_) of five grafting combinations under well-watered and water-deficit stress conditions. Capital letters denote significant differences between the water regimes within each graft combination and lower-case letters denote significant differences among the grafting combinations within each water regime according to the LSD test (*p* ≤ 0.05). Columns are mean ± standard error. Note: HM = non-grafted HM 1823; HM/HM = HM 1823 self-grafted; HM/MU = HM 1823 grafted onto Multifort; HM/PN = HM 1823 grafted onto *S. pennellii*; and HM/PR = HM 1823 grafted onto *S. peruvianum*.

Significant declines in net photosynthetic rates were observed for all grafting and non-grafted combinations under water-deficit stress (Figure 4A). Under that stress condition, plants grafted on *S. pennellii* and *S. peruvianum* rootstocks showed approximately a 75% net photosynthetic rate reduction compared to their well-watered controls. However, the other grafting combinations showed reductions of up to 92% in the net photosynthetic rate when subjected to water-deficit stress. Interestingly, this reduction was almost 100% in non-grafted HM plants, indicating the relative positive effect of grafting.

Eight days after water withholding, all grafting combinations also showed a significant reduction in stomatal conductance compared to their well-watered controls (Figure 4B). The reduction between the two water regimes was approximately 91%, 90%, 89%, 78%, and 86% for HM, HM/HM, HM/MU, HM/PN, and HM/PR, respectively. Under well-watered conditions, the stomatal conductance of HM/PR (0.480 mmol H_2_O m^−2^ s^−1^) was higher than the other grafting combinations. In the water-deficit stress condition, all grafting combinations had the equivalent stomatal conductance values.

Environmental conditions influence stomatal functions, with stomatal closure being one of the first plant responses to water deficit [36,37]. Tomato landraces showed that soil moisture depletion results in an 86% reduction in stomatal conductance to avoid excessive water loss [7].

Stomatal conductance controls both the CO_2_ influx and water efflux in plants [38,39,40]. Stomatal closure deprives leaves of CO_2_, decreasing the photosynthetic carbon assimilation in favor of photorespiration [15]. Rapid stomatal closing was a drought-induced characteristic for *S. pennellii* and other wild-type tomato species [41]. In non-grafted and self-grafted pepper plants under drought stress, there was a drastic decline of net photosynthesis as a result of stomatal closure compared to other grafting combinations [42]. These authors indicated that this tolerance in water stress exhibited by the grafted pepper plants with rootstocks is in part related to the limited oxidative stress with decreased H_2_O_2_ concentration and diminished membrane lipid peroxidation. However, the use of tolerant rootstock reduces the effects of water deficit on photosynthetic activity. For example, in [43], it was shown that plants grafted onto *S. mammosum* improved net photosynthesis compared to self-grafted tomatoes under drought stress. It has also been reported that the *S. habrochaites* introgression line renders scions more tolerant to water-deficit stress through a better stomatal regulation [44]. Luffa rootstock-grafted cucumber showed a higher CO_2_ assimilation rate and lower transpiration rate than self-grafted plants under water-deficit conditions, consequently increasing the WUE and demonstrating that the grafted plants are more efficient in terms of the use of water [45]. A study [16] observed that plants grafted onto the tolerant rootstocks, despite the reduction in the photosynthetic rate, maintained the protective capacity of the photosynthetic machinery mediated by osmotic adjustment (based on higher proline content).

#### 2.2.1. Instantaneous and Intrinsic Leaf Water Use Efficiency

**Figure 5:**
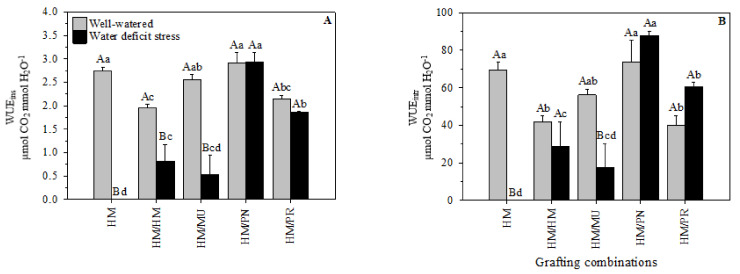
(**A**) Instantaneous leaf water use efficiency (WUE_ins_) and (**B**) intrinsic leaf water use efficiency (WUE_intr_) of five grafting combinations under well-watered and water-deficit stress conditions. Capital letters denote significant differences between water regimes within each graft combination and lower-case letters denote significant differences among grafting combinations within each water regime according to the LSD test (*p* ≤ 0.05). Columns are mean ± standard error. Note: HM = non-grafted HM 1823; HM/HM = HM 1823 self-grafted; HM/MU = HM 1823 grafted onto Multifort; HM/PN = HM 1823 grafted onto *S. pennellii*; and HM/PR = HM 1823 grafted onto *S. peruvianum*.

Instantaneous water use efficiency (WUE_ins_) significantly decreased under water-deficit conditions in HM, HM/HM, and HM/MU combinations by 100%, 58.38%, and 79.1%, respectively, compared to the well-watered condition (Figure 5A). The highest WUE_ins_ value was observed in HM/PN (2.92 µmol CO_2_ mmol H_2_O^−1^) and HM/PR (1.856 µmol CO_2_ mmol H_2_O^−1^). The same trend was observed for WUE_intr_, except for HM/HM, in which no significant difference was observed between the two water regimes. HM/PN (87.79 µmol CO_2_ mmol H_2_O^−1^) and HM/PR (60.60 µmol CO_2_ mmol H_2_O^−1^) also showed the highest values for WUE_intr_ under water-deficit stress (Figure 5B).

WUE is an important indicator of drought-tolerant species; thus, it is considered a critical physiological parameter for genotype screening [46]. Previous reports indicated that *S. pennellii* could increase the WUE from 25% to 100% under water-deficit conditions as compared to cultivated tomatoes [41]. An increase in the WUE can result from the ABA-mediated reduction of stomatal conductance and by limiting water loss, representing a rootstock-specific trait regulation by root-to-shoot hormonal communications [47].

Tomato plants grafted onto recombinant inbred lines derived from a cross between the cultivated tomato *S. lycopersicum* and the wild species *S. pimpinellifolium* presented high agronomic WUE and maintained tomato yield when compared to self-grafted plants [47].

In our study, plants grafted onto *S. pennellii* and *S. peruvianum* displayed the lowest net photosynthetic rate reductions compared to the other grafting combinations, which led to high WUE_ins_ and WUE_intr_ under water-deficit conditions. Therefore, these results suggest much lower water requirements under water-deficit conditions when using these wild-type rootstocks.

#### 2.2.2. Lipid Peroxidation and Amino Acids

**Figure 6:**
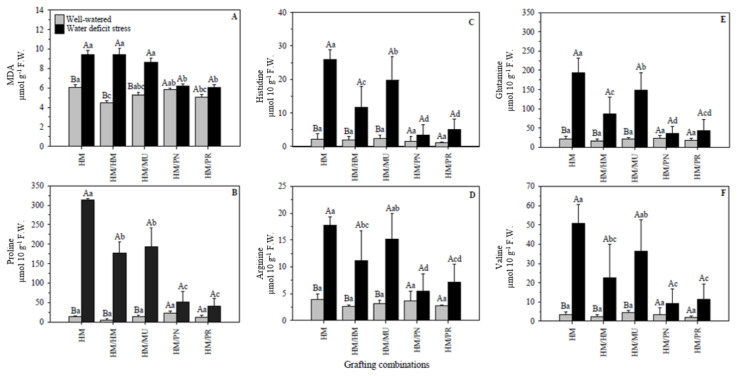
(**A**) Malondialdehyde (MDA) and (**B**) proline, (**C**) histidine, (**D**) arginine, (**E**) glutamine, and (**F**) valine of five grafting combinations under well-watered and water-deficit stress conditions. Capital letters denote significant differences between the water regimes within each graft combination and lower-case letters denote significant differences among grafting combinations within each water regime according to the LSD test (*p* ≤ 0.05). Columns are mean ± standard error. Note: HM = non-grafted HM 1823; HM/HM = HM 1823 self-grafted; HM/MU = HM 1823 grafted onto Multifort; HM/PN = HM 1823 grafted onto *S. pennellii*; and HM/PR = HM 1823 grafted onto *S. peruvianum*.

Compared to their respective controls, significant changes in the malondialdehyde (MDA) content as induced by the water deficit were observed (Figure 6A). Water deficit caused a significant rise in the MDA content of HM (35.8%), HM/HM (52.8%), and HM/MU (38.4%), but did not affect HM/PN and HM/PR. Under stress conditions, the lowest MDA content was measured in HM/PN (6.17 µmol g^−1^ F.W.) and HM/PR (6.08 µmol g^−1^ F.W.).

Similar to other abiotic stresses, water deficit increases the formation of reactive oxygen species (ROS) in plants, causing the accumulation of free radicals of active oxygen, which leads to the oxidation of the cell membrane, inducing lipid peroxidation. The final product of lipid peroxidation is MDA [48,49,50,51].

MDA content has been used to measure the degree of cell damage and as an indicator of water-deficit stress [51,52]. Previous studies have shown that low MDA content is associated with water-stress tolerance in tomato plants [32,34,53].

Tomato plants grafted onto ‘Zarina’ rootstock had low MDA content under water deficit, suggesting that these plants can maintain cellular homeostasis, which confers tolerance to water deficit [54]. Our results showed that the lowest MDA content recorded in HM/PN and HM/PR under water deficit confirmed that *S. pennellii* and *S. peruvianum* rootstocks play an essential role in mitigating the oxidative damage caused by water deficit.

The accumulation of osmolytes, such as amino acids, a variety of sugars, sugar alcohols (mannitol, trehalose, and galactinol), quaternary, and other amines (glycine betaine and polyamines), during drought stress stabilize proteins and membranes, and reduce osmotic potential to prevent cellular dehydration [55]. Amino acids, in particular, are involved in a plethora of cellular functions influencing several physiological processes such as plant growth and development, intracellular pH control, the generation of metabolic energy, or redox power during abiotic stresses [56]. Although several studies investigated the drought-related accumulation of total amino acids due to proteolysis, very few studies have focused on the accumulation of specific amino acids in tomatoes. Significant differences were observed in most commonly induced osmolytes, such as proline, histidine, arginine, glutamine, and valine contents, in the leaves of both grafting combinations under water-deficit conditions. The interaction of water regimes and grafting combinations was not significant for aspartic acid (*p* = 0.13), glutamic acid (*p* = 0.09), citrulline (*p* = 0.83), tyrosine (*p* = 0.41), GABA (*p* = 0.18), methionine (*p* = 0.30), isoleucine (*p* = 0.19), leucine (*p* = 0.12), phenylalanine (*p* = 0.06), and glutathione (*p* = 0.22) (data not shown).

Proline, histidine, arginine, glutamine, and valine analysis in leaves revealed significant differences between the water regimes with the highest values recorded in water-deficit plants (Figure 6B–F). However, no such significant differences were observed for HM/PN and HM/PR when both water regimes were compared. HM showed the highest proline, histidine, arginine, glutamine, and valine contents when compared to self-grafted HM/HM plants under water-deficit conditions. This indicates the importance of grafting in providing better osmotic adjustment, as they do not require much of an increase in proline or other amino acids. HM/PN and HM/PR presented the lowest proline (50.89 and 40.41 µmol 10 g^−1^ F.W., respectively), histidine (3.46 and 5.07 µmol 10 g^−1^ F.W., respectively), arginine (5.41 and 7.18 µmol 10 g^−1^ F.W., respectively), glutamine (35.30 and 43.66 µmol 10 g^−1^ F.W., respectively), and valine (9.09 and 11.47 µmol 10 g^−1^ F.W., respectively) contents when the water-deficit stress was imposed.

Osmolytes buffer the cytosolic pH and maintain both cell redox status and cell turgor in plant tissues. Drought drives the accumulation of specific amino acids [57]. In our study, proline was the amino acid more clearly induced by drought in HM, HM/HM, and HM/MU, increasing significantly by >15-folds. Proline is recognized as the most critical metabolite, playing a vital role in the metabolism and physiology of plants under stress [58]. High abundant amino acids such as proline, arginine, asparagine, glutamine, and GABA are synthesized during abiotic stress [59], while the accumulation of low abundant amino acids results from protein degradation. Under osmotic stress conditions, proline has diverse roles beyond protecting cellular functions through ROS-scavenging, such as that of stabilizing proteins, membranes, and subcellular structures [60,61,62].

Proline accumulation has been reported to occur after plants are exposed to abiotic stress [63]. In several studies, the higher proline accumulation under stress conditions has been proposed to serve as a biochemical marker to identify stress-tolerant genotypes [64].

Metabolic adjustments are a generalized response in many plants [65] but they only represent part of the stress-signaling mechanism that influences adaptive responses. Wild tomato species such as *S. pennellii* accumulates organic solutes such as diamine putrescine and *S. peruvianum* accumulates total carbohydrates [32,65,66] if inorganic solutes are insufficient to reduce the osmotic potential. The proline content of tomato leaves has been modulated according to the nutrient concentration and relative water content of leaves [67]. In our experiment, the RWC of grafted plants using wild-rootstocks (HM/PN or HM/PR) was higher than the ungrafted or self-grafted plants, implying that the induction of proline, or otherwise an energy-intensive process, is circumvented in these plants due to higher RWC.

Unlike the reports of the grafted tomato plants using cultivated rootstocks [68] or pepper plants [16], our study shows that the wild rootstocks do not directly impact deficit-induced proline accumulation in the scion. Consistent with our results, no significant changes in the proline content during salt stress in *Solanum pennellii* and *Solanum peruvianum*, as well as during drought stress in *S. peruvianum*, have been reported [69]. It has been suggested that the osmotic adjustment using proline is not the only strategy used to maintain turgor in plants [34].The drought-tolerant tomato cv. Zarina accumulated lower proline content than the sensitive genotypes [34]. Allan et al. [70] reported that higher tolerance in tomato plants to water stress was not correlated with higher osmotic adjustment. Conversely, it was observed that water deficit-tolerant wheat plants showed a high correlation of RWC related to low proline concentration [71].

Our data showed the impact of rootstocks on the photosynthetic and biochemical parameters of tomato plants. The use of a common scion (HM) allowed us to compare the performance of rootstocks across grafting combinations. The genotype of rootstocks largely influences the rooting and grafting capacity, abiotic and biotic stress tolerance, and scion phenotypes. Rootstocks affect the scion performance by regulating the translocation of hormones; nutritional components, such as amino acids, salts, organic solutes, etc.; and genetic elements, such as RNA species through the phloem [72]. Furthermore, the grafted tomato plants’ morphological and physiological changes were attributed to altered gene expression due to the eggplant rootstock [72].

## 3. Materials and Methods

### 3.1. Plant Material and Grafted Seedling Production

The experiment was conducted in a greenhouse at the Texas A&M AgriLife Research Center at Uvalde, TX (29.21° N, 99.79° W), from February to June 2018.

The tomato cultivar ‘HM 1823’ (Harris Moran Seed Company, Modesto, CA, USA) was either self-grafted or grafted onto the following rootstocks: LA0716 (*Solanum pennellii*), PI128659 (*Solanum peruvianum*), and ‘Multifort’ (De Ruiter Seeds Inc., Lakewood, CO, USA).

‘Multifort’ is a commercially available interspecific tomato hybrid rootstock (*S. lycopersicum* × *S. habrochaites*) and was selected as one of the most representative commercial rootstocks used in the United States of America [73]. The two accessions, *S. pennellii* and *S. peruvianum*, were included as rootstock due to their reported tolerance to water stress [21]. Seeds of the two accessions were obtained from the UC Davis/C.M. Rick Tomato Genetics Resource Center and were maintained by both the Department of Plant Sciences, University of California, Davis, CA 95616 (TGRC, http://tgrc.ucdavis.edu/)(accessed on 18 September 2017), and the U.S. National Germplasm System (GRIN, http://npgsweb.ars-grin.gov) (accessed on 15 September 2017).

Seeds of the rootstocks *S. peruvianum*, *S. pennellii*, and ‘Multifort’ were sown into styrofoam flats containing a commercial soilless media at 17, 11, and 2 days, respectively, prior to the scion HM 1823. The time interval between the sowing was necessary to match the stem diameter sizes of the scion and rootstock, and to obtain a higher percentage of graft survival.

Plants were grafted at the 3–4 leaf stage 26 days after scion emergence using the splice grafting method.

Grafted plants were placed in a healing chamber with high relative humidity (between 85% and 95%) at 22 °C in darkness for the first 3 days. From the fourth day through to the eighth day, dim light and reduced relative humidity were introduced by gradually opening the healing chamber. Healed plants were moved to the greenhouse after 8 days, where they were further hardened off for 3 to 4 days before the beginning of the pot experiment.

### 3.2. Experimental Design and Growth Conditions

The experiment was designed as a factorial combination of two irrigation treatments (well-watered and water deficit) and five grafting combinations (self-grafted ‘HM 1823’/‘HM 1823’(HM/HM), ‘HM 1823’ grafted onto *S. pennellii* (HM/PN), ‘HM 1823’ grafted onto *S. peruvianum* (HM/PR), and ‘HM 1823’ grafted onto ‘Multifort’ (HM/MU), as well as non-grafted ‘HM 1823’ (HM)). The treatments were arranged in a randomized complete block design with three replications of four plants each.

Fully healed and hardened-off grafted plants and non-grafted controls were transplanted into 10 L pots filled with sandy loam soil (sand:silt: clay; 77:8:15%). Soil moisture sensors (ECH_2_O^®^ soil moisture probes, Em5b, Decagon Devices Inc., Pullman, WA, USA) were placed in the pots to monitor the volumetric water content continuously.

One month after transplanting, the two irrigation treatments were initiated. In the water-deficit conditions, irrigation was withheld for 8 days, at which point-strong midday-wilting was evident, marking the end of the dry-down cycle. Pots were weighed daily at 8:00 a.m. and water was added daily only to well-watered-condition pots to maintain the pot weight at the container capacity, which is equivalent to 0.45 m^3^ m^−3^.

Non-grafted and grafted plants were fertilized using a 20N-5P-16K fertilizer and micronutrients during the growth period. Plants were individually staked in each pot to keep the vine upright.

The gas exchange measurements, determination of plant water status, and leaf samples for the biochemical analysis were taken and performed on the 8th day at the end of the water withholding period.

### 3.3. Plant Water Status Determination

Plant water relations were determined by measurements of the leaf water potential (Ψ_w,_ MPa) and relative water content (RWC, %). The penultimate leaflet of the fourth leaf from the apex to the base was cut at petiole insertion and immediately placed in the pressure chamber instrument (Model 615, PMS Instrument Company, Albany, OR, USA) for water potential measurements.

The relative water content was determined using four composite leaf discs (1 cm diameter) collected from similar mature leaflets located at the plants’ base, middle, and apex. Fresh weight (FW) was obtained by weighing the leaf discs immediately after harvesting. Then, leaf discs were submerged in distilled water in darkness for 24 h to determine the turgid weight (TW). Subsequently, the discs were dried in an oven at 65 °C for 48 h to obtain the dry weight (DW). The RWC of the composited leaf sample was calculated using the following equation:


(1) $$\mathrm{RWC}\;\left(\%\right)\;=\;\left(\frac{\mathrm{FW}\;-\;\mathrm{DW}}{\mathrm{TW}\;-\;\mathrm{DW}}\right)\;*\;100\;$$

### 3.4. Gas Exchange Measurements

The net photosynthetic rate (A_N_, µmol CO_2_ m^−2^ s^−1^), stomatal conductance (g_s_, mol H_2_O m^−2^ s^−1^), and leaf transpiration rate (E, mmol H_2_O m^−2^ s^−1^) were measured in the penultimate leaflet of the third fully expanded leaf, counted from the apex [74]. Gas exchange was measured with a portable photosynthesis system LI-6400 XT fitted with a 6 cm^2^ leaf cuvette (LI-COR Biosciences, Lincoln, NE, USA). The measurements were performed between 10:00 and 12:00 a.m.

### 3.5. Instantaneous and Intrinsic Leaf Water Use Efficiency Calculation

Instantaneous leaf water use efficiency (WUE_ins_, mol CO_2_ mmol^−1^ H_2_O) was calculated as the ratio between A_N_ and E, and intrinsic leaf water use efficiency (WUE_intr_, µmol CO_2_ mmol H_2_O^−1^) was calculated as the ratio between A_N_ and g_s_ [75].

### 3.6. Lipid Peroxidation Determination

Lipid peroxidation was evaluated as an indicator of cell membrane damage by measuring malondialdehyde (MDA, µmol g^−1^ F.W.) [76]. The penultimate leaflet of the third leaf from the apex was harvested, immediately frozen in liquid nitrogen, and stored at −80 °C until use for MDA determination.

Frozen leaves were thawed and then 0.1 g were homogenized in 0.5 mL of 1% (*m*/*v*) trichloroacetic acid (TCA) solution. The homogenate was centrifuged at 15,000× *g* for 10 min at 4 °C and 1.5 mL of 20% TCA containing 0.5% (*m*/*v*) thiobarbituric acid (TBA) was added to the supernatant aliquot. The mixture was heated at 95 °C for 25 min and then quickly chilled on ice. The absorbance of the solution was recorded at 532 and 600 nm after centrifugation. The MDA concentration was calculated using the equation described below:


(2) $$\mathrm{MDA}\;\left({\mu \mathrm{mol}\;g}^{-1}\right)\;=\;\frac{\left(A532\;-\;A600\right)}{155}\;*\;1000$$

### 3.7. Amino Acids Extraction and Derivatization

Penultimate leaflets of the third leaf from the apex were harvested, immediately frozen in liquid nitrogen, and stored at −80 °C until the extraction of amino acids (µmol g^−1^ F.W.). Frozen plant tissue (~20 mg) was homogenized into a fine powder using 3 mm Demag stainless steel balls (Abbott Ball Company, West Hartford, CT, USA, USA) and a Harbil model 5G-HD paint shaker. Homogenized tissue was suspended in 20 mM of cold HCl (10 μL per mg of tissue), incubated on ice for 20 min, and centrifuged at 14,609× *g* for 20 min at 4 °C. The supernatant was filtered using 0.45 uM 96-well filters (Pall Life Sciences, Ann Arbor, MI, USA) and derivatized with the AccQ-Tag Ultra-Fluor™ derivatization kit (Waters Corporation, Milford, MA, USA). For derivatization, 5 μL of plant extracts were mixed with 35 μL of borate buffer and 10 μL of the AccQ•Tag Ultra-Fluor™ reagent, and the reaction was allowed to proceed for 10 min at 55 °C.

### 3.8. Amino Acids Analysis with Waters UPLC ESI MS-MS

The amino acid analysis of tomato leaves was carried out using the Waters UPLC-ESI-MS/MS platform on a Waters Acquity H-class UPLC system coupled to a Waters Xevo TQs mass spectrometer using an electrospray ionization (Waters, Milford, MA, USA) (ESI) probe. The instrumentation method and separation gradient were standardized based on previously established protocols [77,78]. In brief, one microliter of the derivatized sample was injected for analysis using the Water’s AccQ•Tag Ultra column with 60 °C set as the column heater temperature. The mobile phase was composed of water (0.1% formic acid *v*/*v*) (A) and acetonitrile (0.1% formic acid *v*/*v*) (B), and the mobile phase flow rate was maintained at 0.6 mL/min. The non-linear separation gradient was maintained at 0–1.5 min (96% A), 3.0 min (95.0% A), 5 min (92% A), 5.10 min (72% A), and 6.10 min (5% A). Data integration and quantitation were performed using the Waters Target Links^TM^ software (Masslynx 4.1) and the amounts of amino acids were quantified based on the Water 24 amino acids standard mix.

### 3.9. Statistical Analysis

Data were analyzed using a two-way ANOVA and mean differences among the treatments were analyzed using Ficher’s Least Significant Difference (LSD) at *p* < 0.05. The statistical analysis was performed using the ExpDes.pt package from the R program (Version 3.4.1).

## 4. Conclusions

This study evaluated whether tomato plants grafted onto *S. pennellii* and *S. peruvianum* were more efficient and had less oxidative damage than non-grafted plants under water-deficit stress. The results of this investigation demonstrated that the wild-type rootstocks influenced the physiological and biochemical responses of tomato plants under water-deficit stress conditions. The grafting combinations with *S. pennellii* (HM/PN) and *S. peruvianum* (HM/PR) showed the highest RWCs; a lesser photosynthetic rate reduction under water-deficit stress; and higher efficiency in leaf water use. Tomato plants grafted onto wild-type *S. pennellii* and *S. peruvianum* rootstocks were efficient in terms of water use, with minimal oxidative damage under water-deficit stress.

## Figures and Tables

**Table 1:** Summary of the two-way analysis of variance with *p*-values for the effects of irrigation treatments (IT), grafting combinations (GC), and ITxGC interactions on water potential (Ψ_w_), relative water content (RWC), net photosynthetic rate (A_N_), stomatal conductance (g_s_), leaf transpiration rate (E), instantaneous leaf water use efficiency (WUE_ins_), intrinsic leaf water use efficiency (WUE_intr_), malondialdehyde (MDA), proline, histidine, arginine, glutamine, and valine.

Response	SV ^1^	DF ^2^	*p*-Value	CV (%) ^3^
Ψ_w_ (MPa)	Irrigation treatments (IT)	1	<0.001	6.52
	Grafting combinations (GC)	4	<0.001	
	ITxGC	4	<0.001	
RWC (%)	Irrigation treatments (IT)	1	<0.001	6.21
	Grafting combinations (GC)	4	0.478	
	ITxGC	4	<0.001	
A_N_ (µmol CO_2_ m^−2^ s^−1^)	Irrigation treatments (IT)	1	<0.001	21.87
	Grafting combinations (GC)	4	0.001	
	ITxGC	4	0.022	
g_s_ (mmol H_2_O m^−2^ s^−1^)	Irrigation treatments (IT)	1	<0.001	45.38
	Grafting combinations (GC)	4	0.003	
	ITxGC	4	0.035	
E (mmol H_2_O m^−2^ s^−1^)	Irrigation treatments (IT)	1	<0.001	23.96
	Grafting combinations (GC)	4	<0.001	
	ITxGC	4	0.113	
WUE_ins_ (µmol CO_2_ mmol H_2_O^−1^)	Irrigation treatments (IT)	1	<0.001	21.91
	Grafting combinations (GC)	4	<0.001	
	ITxGC	4	<0.001	
WUE_intr._ (µmol CO_2_ mmol H_2_O^−1^)	Irrigation treatments (IT)	1	<0.001	30.63
	Grafting combinations (GC)	4	<0.001	
	ITxGC	4	<0.001	
MDA (µmol g^−1^ F.W.)	Irrigation treatments (IT)	1	<0.001	9.10
	Grafting combinations (GC)	4	<0.001	
	ITxGC	4	<0.001	
Proline (µmol 10 g^−1^ F.W.)	Irrigation treatments (IT)	1	<0.001	42.18
	Grafting combinations (GC)	4	<0.001	
	ITxGC	4	<0.001	
Histidine (µmol 10 g^−1^ F.W.)	Irrigation treatments (IT)	1	<0.001	46.22
	Grafting combinations (GC)	4	<0.001	
	ITxGC	4	<0.001	
Arginine (µmol 10 g^−1^ F.W.)	Irrigation treatments (IT)	1	<0.001	40.72
	Grafting combinations (GC)	4	0.006	
	ITxGC	4	0.0125	
Glutamine (µmol 10 g^−1^ F.W.)	Irrigation treatments (IT)	1	<0.001	43.56
	Grafting combinations (GC)	4	<0.001	
	ITxGC	4	<0.001	
Valine (µmol 10 g^−1^ F.W.)	Irrigation treatments (IT)	1	<0.001	63.25
	Grafting combinations (GC)	4	0.003	
	ITxGC	4	0.006	

^1^ SV: source of variation; ^2^ DF: degrees of freedom; ^3^ CV: coefficient of variation.

## Data Availability

Data is contained within the article.
